# The mediating role of depressive and anxiety symptoms in the association between obesity and problematic social media use in young adults

**DOI:** 10.1002/osp4.434

**Published:** 2020-06-16

**Authors:** Anna F Jolliff, Megan A Moreno, Jonathan D'Angelo

**Affiliations:** ^1^ Department of Pediatrics University of Wisconsin—Madison Madison Wisconsin USA

**Keywords:** anxiety, depression, obesity, social media

## Abstract

**Background:**

Obesity has been associated with problematic internet use or internet use characterized by impulsivity, dependence, risk taking or impairment. Despite the unique affordances and growing popularity of social media, few studies have investigated obesity in relation to the problematic use of social media in contrast to general internet use.

**Objective:**

The purpose of this study was to explore the relationship between obesity and problematic social media use and to test symptoms of anxiety and depression as potential mediators of this relationship.

**Methods:**

A cross‐sectional online survey was administered to young adults between the ages of 18–25 using the Qualtrics platform. Two mediation models were tested using model 4 of the PROCESS Macro in SPSS.

**Results:**

Participants (*n* = 4939) were between the ages of 18–25 (*M* = 21.74, *SD* = 2.3). Participants were 50.6% female (*n* = 2496) and 58.1% White (*n* = 2871). Reporting obesity was positively associated with reporting increased levels of problematic social media use, *B* = 1.15, *SE B* = .32, *t*(1, 4938) = 3.59, *p* < .001. The indirect effects in each model from obesity to problematic social media use, through both symptoms of anxiety and depression separately, were significant, *B* = .14, *SE B* = .05, confidence interval [0.055, .231] and *B* = .16, *SE B* = .07, confidence interval [0.018, .317], respectively.

**Conclusions:**

Young adults who present both with obesity and with symptoms of depression or anxiety are more at risk for problematic social media use. These patients may benefit from education on health‐promoting social media use.

## 1 INTRODUCTION

People with obesity are at higher risk of problematic internet use (PIU) or internet use that is characterized by impulsivity, dependence, risk taking or impairment.^1^, ^2^, ^3^ However, despite its unique affordances and broad uptake, few studies have investigated how obesity may relate to problematic *social media* use (PSMU), in particular.^4^, ^5^ Social media use is different from internet use in that it affords possibilities for synchronous and asynchronous communication, self‐presentation and self‐disclosure.^6^ Further, it is unknown whether the observed link between obesity and PIU may be explained in part by mental health concerns, which occur at higher rates in those who have obesity.^7^

In contrast to research on PSMU (often called ‘social media addiction’), research on PIU (often called ‘internet addiction’) is more robust. Young's seminal work on internet addiction has been cited in over 1000 studies.^8^ At its core, PIU differs from PSMU insofar as it can pertain to any online platform—not just social networking sites. PSMU may include using social media to forget personal problems or trying unsuccessfully to cut down on the use of social media.^9^ In contrast, PIU is broader; it may include, for example, viewing inappropriate material online or experiencing a lack of control while engaging with any web‐based content.^2^ Both PIU and PMSU may be significant when they result in impairment (e.g., a failure to maintain relationships offline) or physical impairment (e.g., using the internet or social media specifically at the expense of sleep or other health‐promoting behaviours).^2^ Although research has shown positive associations between obesity and PIU,^1^, ^10^ the same link is less documented for PSMU.

Both physiological and psychological mechanisms have been suggested to explain the link between obesity and PIU. Some have suggested that, because internet use is a largely sedentary pastime and is associated with less healthy food consumption, it may contribute to obesity.^11^ To prevent obesity, parents have been advised to promote more offline recreational activities in their children.^10^ Tabatabaee and colleagues suggest that heavy internet use leads to obesity indirectly through poor sleep quality, lack of physical activity and more consumption of fast food.^12^ Although the aforementioned research^10^, ^11^, ^12^ suggests that obesity is related to PIU through lifestyle factors, it is unclear whether obesity is predictive of PSMU for the same reasons.

There is also the potential for psychological pathways to explain the relationship between obesity and PIU. Symptoms of depression and anxiety have been associated with PIU.^13^ Additionally, obesity has been associated with symptoms of depression^14^, ^15^ and anxiety.^16^ These mental health symptoms may provide a pathway through which obesity predicts PIU. People with depressive and anxiety symptoms may be more likely to go online to alleviate or distract themselves from negative affect.^17^ Those with obesity have also endorsed experiential avoidance at elevated rates, and spending excessive time online may be one such manifestation of avoidance.^18^ People with depressive symptoms may desire to escape feelings of guilt, worthlessness or fatigue, whereas those with anxiety symptoms may seek to distract themselves from nervousness or worry.^19^ In a culture that stigmatizes larger bodies, those who concurrently have obesity *and* depressive or anxious symptoms may find relief or escape in media use. If these explanations are accurate, a similar association between obesity and PMSU could exist.

In contrast to PIU, the relationship between PSMU, mental health and physical health has received less attention.^18^, ^19^, ^20^, ^21^, ^22^ This is an important gap, given that social media is theoretically and practically distinct from other forms of technology. Social media has unique affordances, such as promoting connection, identity formation, self‐expression and social learning.^5^, ^20^ Social media is also used as a means of emotional support, particularly among those with challenging or stigmatized conditions.^21^, ^22^ However, exposure to social media has also been associated with negative outcomes, such as negative self‐comparison and lower self‐esteem.^23^, ^24^ More research is needed to determine if the negative physical and mental health symptoms traditionally associated with other forms of problematic technology use are also associated with PSMU.

The purpose of the present study was to explore the relationship between obesity and PSMU and to test anxiety and depressive symptoms as potential mediators of this relationship. We hypothesized that obesity would be positively associated with PSMU in young adults (H1). We further hypothesize that this relationship would be mediated by symptoms of anxiety (H2) and depression (H3).

## 2 METHODS

A cross‐sectional online survey was administered using the Qualtrics platform in April and May of 2018. Institutional Review Board approval was obtained from the University of Wisconsin—Madison. This study is the first manuscript emerging from a larger project to understand various forms of problematic technology use and the critical health issues with which it has been linked in the young adult population, among them sleep disruption, chronic illness and symptoms of mental illness.

### 2.1 Participants and recruitment

Participants for this study were recruited through Qualtrics, a platform that partners with survey research companies to draw diverse pools of participants. Qualtrics sends survey invitations to existing panel members who meet specific eligibility criteria. Qualtrics sampling has been shown to obtain demographic characteristics such as education level, marital status, political affiliation and annual household income that are within 10% of corresponding values in the US population.^23^ The present study included a sample of English‐speaking young adults between the ages of 18–25 who were racially and ethnically representative of the US census population.^24^ The target sample size was based on conservative estimates of the prevalence of both problematic technology use and depression in this age group, given the aims of the larger project from which these data were drawn.^25^, ^26^ Participants were compensated by Qualtrics for participating. Qualtrics participants agree on the details of compensation before taking any survey; compensation varies by participant and may be in the form of airline or retail points, gift cards or cash.

### 2.2 Survey measures

Participants provided demographic information, including gender, age, ethnicity and race.

### 2.3 Mental health measures

#### 2.3.1 Depressive symptoms

The Patient Health Questionnaire 9 (PHQ‐9)^27^ is a widely used, self‐report, nine‐item measure of depressive symptomatology based on the fourth Diagnostic and Statistical Manual criteria for depression (DSM IV). Patients endorse the frequency with which they have experienced nine symptoms in the past 2 weeks. Example items include ‘difficulty falling or staying asleep’ and ‘feeling down, depressed, or hopeless’. Response options include ‘not at all’, ‘some’, ‘often’ and ‘nearly all the time’. A score of 5–10 indicates mild depression; 10–15 indicates moderate depression; and over 15 indicates severe depression.

#### 2.3.2 Anxiety symptoms

The Generalized Anxiety Disorder Scale—7 (GAD‐7) is a brief, self‐report, seven‐item screener for anxiety based on DSM IV criteria.^28^ Patients indicate the frequency with which they have experienced seven anxiety symptoms in the last 2 weeks. Example items include ‘feeling nervous, anxious, or on edge’ and ‘worrying too much about different things’. Response options include ‘not at all’, ‘several days’, ‘more than half the days’ and ‘nearly every day’. As with the PHQ‐9, scores of 5, 10 and 15 represent cut‐off points for mild, moderate and severe anxiety, respectively.

### 2.4 Obesity

To obtain a categorical measure of obesity, the study team consulted with a health services researcher who has used a similar question in studies of younger populations.^29^ Participants were asked: ‘Have you ever been told by a doctor or healthcare provider that you have any of the following chronic or ongoing health conditions?’ Participants could select one or more of 10 response options, one of which was obesity.

### 2.5 Problematic social media use

The Bergen Social Media Addiction Scale (BSMAS)^30^ is a six‐item scale that measures the problematic use of social media.^27^, ^28^, ^29^ Items represent the core features of addiction explicated by Griffiths in 2005, such as salience (‘spend a lot of time thinking about social media or planning how to use it’), tolerance (‘feel an urge to use social media more and more’) and withdrawal symptoms (‘become restless or troubled if you are prohibited from using social media’).^9^, ^31^ Response options include ‘very rarely’, ‘rarely’, ‘sometimes’, ‘often’ and ‘very often’. No formal cut‐off scores exist, although a score of 19 to indicate risk for social media addiction was proposed in one study.^32^, ^33^

### 2.6 Analysis

The research team hypothesized that obesity would be associated with PSMU (H1) and that this relationship would be mediated by anxiety (H2) and depressive symptoms (H3). In order to assess these hypotheses, two mediation models were tested using model 4 of the PROCESS Macro in SPSS.^34^ Confidence intervals (CIs) were based on 5000 bootstrap samples. The first model tested whether anxiety symptoms mediated the relationship between obesity and PSMU. The second model tested whether depressive symptoms mediated the relationship between obesity and PSMU. For each model, the mediator variable from the other model was controlled for, as anxiety and depression are likely related and highly correlated, and the goal was to isolate the variance uniquely related to each condition. All variables included in these analyses were continuous.

## 3 RESULTS

### 3.1 Demographics

Six thousand participants were surveyed. Of those participants, only those who answered each item relevant in the larger project as well as the present subproject (*n* = 4939) were retained for analysis. Participants with missing data were dropped in a listwise fashion. Participants were between the ages of 18–25 (*M* = 21.74, *SD* = 2.3). Participants were 50.6% female (*n* = 2496) and 58.1% White (*n* = 2871). See Table 1 for complete demographic information.

**TABLE 1:** Demographic information from sample of 18–25 year olds

*n* = 4936–4939	Study sample *M* (*SD*)/*n* (%)
Age	21.74 (2.3)
Obesity	346 (7%)
Gender	
Female	2496 (50.6%)
Male	2283 (46.3%)
Non‐binary	70 (1.4%)
Female to male transgender	77 (1.6%)
Male to female transgender	2 (0.0%)
Other	8 (0.2%)
Ethnicity	
Yes—Hispanic or Latino origin	827 (16.8%)
No—Hispanic or Latino origin	4091 (83.2%)
Race	
American Indian/Alaska Native	142 (2.9%)
Asian	258 (5.2%)
Black/African American	641 (13%)
Caucasian/White	2871 (58.1%)
Hispanic/Latino	405 (8.2%)
Native Hawaiian/Pacific Islander	5 (0.1%)
More than one race selected	574 (11.6%)
Other	35 (0.7%)
Missing	8 (0.2%)

The mean score on the PHQ‐9 was 10.17 (*SD* = 7.77). Approximately 20% (20.4%) of participants had scores reflective of mild depression, 20.7% for moderate depression and 28.7% for severe depression. The mean score on the GAD‐7 was 8.40 (*SD* = 6.12). Approximately 27% (26.6%) of participants had symptoms of mild anxiety, 23.3% for moderate anxiety and 17.8% for severe anxiety. The mean score on the BSMAS was 13.34 (*SD* = 5.72), which is comparable with the mean obtained by scale authors.^31^ Within the sample, 17.9% scored 19 or above.

### 3.2 Main results

To test the hypothesis that obesity would be positively associated with PIU (H1), a simple linear regression was used. As expected, the model was significant, *R* ^2^ = .003, *F*(1, 4937) = 12.92, *p* < .001. Reporting obesity was positively associated with reporting increased levels of PSMU, *B* = 1.15, *SE B* = .32, *t(*1, 4938) = 3.59, *p* < .001.

**FIGURE 1:**
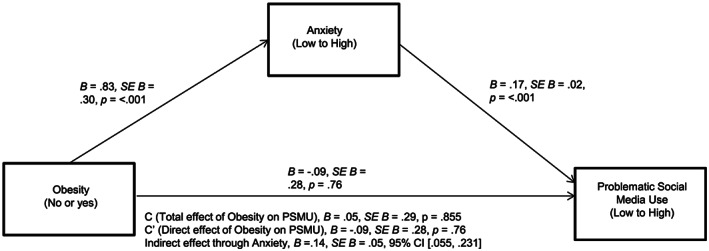
Mediation model with unstandardized coefficients, controlling for effect of depressive symptoms. CI, confidence interval; PSMU, problematic social media use

Hypothesis 2 stated that reporting the chronic condition of obesity would be associated with an increase in self‐reported anxiety symptoms, which in turn would be associated with increased PSMU, while controlling for the effect of depressive symptoms. This indirect effect from obesity to PSMU, through anxiety symptoms, was significant, *B* = .14, *SE B* = .05, CI [0.055, 0.231], providing support to H2. The overall model was significant, *R* ^2^ = .23, *F*(3, 4935) = 481.32, *p* < .001 (see Figure 1 below for the full model with individual pathways). Depressive symptoms were used as a covariate on all downstream variables and were positively associated with anxiety symptoms, *t*(2, 4936) = 77.39, *p* < .001, as well as PSMU, *t*(3, 4935) = 17.57, *p* < .001.

**FIGURE 2:**
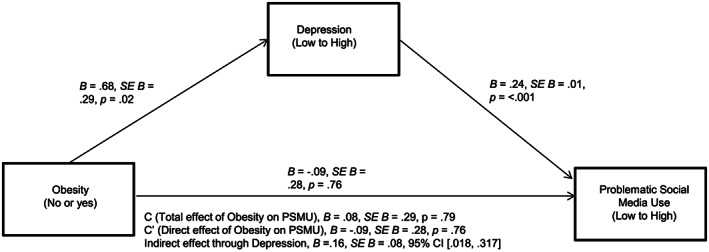
Mediation model with unstandardized coefficients, controlling for effect of anxiety symptoms. CI, confidence interval; PSMU, problematic social media use

Hypothesis 3 stated that reporting the chronic condition of obesity would be positively associated with self‐reported depressive symptoms, which in turn would be associated with PSMU, while controlling for the effect of anxiety symptoms. This indirect effect from obesity to PSMU, through depressive symptoms, was significant, *B* = .16, *SE B* = .08, CI [0.018, 0.317], providing support to H3. The overall model was significant, *R* ^2^ = .23, *F*(3, 4935) = 481.32, *p* < .001 (see Figure 2 for the full model with individual pathways). Anxiety symptoms were used as a covariate and were found to be positively associated with depressive symptoms, *t*(2, 4936) = 77.39, *p* < .001, as well as PSMU, *t*(3, 4935) = 9.47, *p* < .001.

## 4 DISCUSSION

The present study found a significant positive association between obesity and PSMU. Further, this study found that this relationship was mediated by both anxiety and depressive symptoms individually. These findings suggest that the relationship between obesity and PSMU could be at least partly explained by individuals' mental health status.

The present study found a positive association between obesity and technology use. However, in a departure from previous literature, this study examined obesity in relation to PSMU, specifically. Findings suggest that obesity is positively associated with PSMU. Previous research has suggested that the link between PIU and obesity is explained by the sedentary nature of internet use, and the same reasoning could theoretically apply to obesity and PSMU.^3^ Particularly when sedentary activities are reinforcing—in just the way that social media often is—the behaviour may be more likely to increase over time. This, in turn, could cyclically contribute to disrupted sleep schedules, sedentary lifestyles and weight gain.^35^ It is also possible that impulsivity, which is shown to be higher among those with obesity, explains the link between obesity and PSMU.^36^ In this case, impulsivity—a characteristic predictive of maladaptive gambling, higher nicotine use and binge eating, for example—could be predictive of PSMU as well.^37^, ^38^ Future research should continue to examine whether the link between obesity and PSMU is unique from that between obesity and other forms of PIU. This may allow both clinicians and community members to better understand the spectrum of internet use and associated risks to health.

Findings also supported mental health variables as potential mediators of the relationship between obesity and PSMU. Anxiety and depressive symptoms were independently associated with both obesity and PSMU and mediated the link between the two. It may not be the behaviours associated with obesity itself that predict PSMU, but rather characteristics of young adults with anxiety or depressive symptoms who also have obesity. Those with anxiety or depressive symptoms may struggle to find the courage or motivation to leave the house and pursue social interaction, and this may be particularly stressful for those who concurrently have a stigmatized health condition like obesity.^39^ For these young adults, social media could provide a welcome and safe opportunity for social engagement. It is also worth noting that those who have obesity are more at risk for anxiety and depression in general, and there is much work to be done in understanding the particular vulnerabilities of this group.^40^

This study had several limitations. First, participants were recruited online and thus may have used social media at higher rates. However, our sample showed rates of PSMU comparable with other groups.^31^ Second, the design was cross‐sectional, and as such, causal relationships cannot be identified. Longitudinal research should seek to identify whether obesity precedes or follows the onset of PSMU. Third, symptoms of anxiety and depression often co‐occur. However, in the present study, these constructs were examined separately in an attempt to parse out differences in their relation to other study variables. This may limit the ecological validity of findings. Fourth and perhaps most importantly, the measurement of obesity was both conservative and potentially incomplete. Participants were asked ‘Have you ever been told by a doctor or healthcare provider that you have any of the following chronic or ongoing health conditions?’ and obesity was a response option. This phrasing may leave out people with obesity who have not received this diagnosis from a healthcare provider or misidentify people who previously were diagnosed with obesity but had since lost weight. This item may explain why obesity occurred in a relatively small subset of our sample. Last, internalized weight stigma was not measured, which may predict negative affect or PSMU to a greater extent than a diagnosis of obesity.^41^, ^42^, ^43^

Findings from the present study suggest a complex interrelationship between obesity, mental health status and social media use among young adults. Physicians should note that young adults who have obesity and symptoms of mental illness are at a greater risk of PSMU. Thus, strategies for pro‐wellness social media engagement balanced with healthy offline relationships should be discussed. Young adult patients who present with obesity and symptoms of depression or anxiety may benefit from interventions towards health‐promoting social media use.

## CONFLICT OF INTEREST

The authors declared no conflict of interest.
